# Protein–Protein Interaction between Surfactant Protein D and DC-SIGN *via* C-Type Lectin Domain Can Suppress HIV-1 Transfer

**DOI:** 10.3389/fimmu.2017.00834

**Published:** 2017-07-31

**Authors:** Eswari Dodagatta-Marri, Daniel A. Mitchell, Hrishikesh Pandit, Archana Sonawani, Valarmathy Murugaiah, Susan Idicula-Thomas, Béatrice Nal, Maha M. Al-Mozaini, Anuvinder Kaur, Taruna Madan, Uday Kishore

**Affiliations:** ^1^Department of Life Sciences, College of Health and Life Sciences, Brunel University London, Uxbridge, United Kingdom; ^2^Clinical Sciences Research Laboratories, Warwick Medical School, University Hospital Coventry and Warwickshire Campus, Coventry, United Kingdom; ^3^Department of Innate Immunity, National Institute for Research in Reproductive Health, Indian Council of Medical Research, Mumbai, India; ^4^Institute of Environment, Health and Societies, Brunel University London, Uxbridge, United Kingdom; ^5^Department of Infection and Immunity, King Faisal Specialist Hospital and Research Centre, Riyadh, Saudi Arabia

**Keywords:** surfactant protein D, DC-SIGN, HIV-1 infection, protein–protein interactions, gp120

## Abstract

Surfactant protein D (SP-D) is a soluble C-type lectin, belonging to the collectin (collagen-containing calcium-dependent lectin) family, which acts as an innate immune pattern recognition molecule in the lungs at other mucosal surfaces. Immune regulation and surfactant homeostasis are salient functions of SP-D. SP-D can bind to a range of viral, bacterial, and fungal pathogens and trigger clearance mechanisms. SP-D binds to gp120, the envelope protein expressed on HIV-1, through its C-type lectin or carbohydrate recognition domain. This is of importance since SP-D is secreted by human mucosal epithelial cells and is present in the female reproductive tract, including vagina. Another C-type lectin, dendritic cell (DC)-specific intercellular adhesion molecule-3-grabbing non-integrin (DC-SIGN), present on the surface of the DCs, also binds to HIV-1 gp120 and facilitates viral transfer to the lymphoid tissues. DCs are also present at the site of HIV-1 entry, embedded in vaginal or rectal mucosa. In the present study, we report a direct protein–protein interaction between recombinant forms of SP-D (rfhSP-D) and DC-SIGN *via* their C-type lectin domains. Both SP-D and DC-SIGN competed for binding to immobilized HIV-1 gp120. Pre-incubation of human embryonic kidney cells expressing surface DC-SIGN with rfhSP-D significantly inhibited the HIV-1 transfer to activated peripheral blood mononuclear cells. *In silico* analysis revealed that SP-D and gp120 may occupy same sites on DC-SIGN, which may explain the reduced transfer of HIV-1. In summary, we demonstrate, for the first time, that DC-SIGN is a novel binding partner of SP-D, and this interaction can modulate HIV-1 capture and transfer to CD4^+^ T cells. In addition, the present study also reveals a novel and distinct mechanism of host defense by SP-D against HIV-1.

## Introduction

Surfactant protein D (SP-D) is a collagen-containing C-type lectin, belonging to the collectin family (1). The primary structure of human SP-D is composed of three subunits of polypeptide chains; each subunit consists of a short N-terminal region, a triple-helical collagen-like region, an α-helical coiled-coil neck region, and a calcium-dependent highly conserved C-type lectin or carbohydrate recognition domain (CRD) at the C-terminus (2, 3). The primary structure can get cross-linked *via* the cysteine-containing N-terminal region to give rise to a cruciform structure, which can undergo further multimerization to yield a fuzzy ball, where the CRD regions are facing toward the exterior. SP-D, *via* its homotrimeric CRD region, is known to interact with a wide range of viral, bacterial, and fungal pathogens and bring about clearance mechanisms that involve aggregation or agglutination, opsonization, enhanced phagocytosis, and super-oxidative burst (3, 4). Primarily found in the lungs as a part of pulmonary surfactant, SP-D has been localized at a range of extrapulmonary sites as a part of mucosal defense system (5).

SP-D is present throughout the female genital tract, with likely involvement in the prevention of uterine infections (6). Epithelial linings of vagina, cervix, uterus, fallopian tubes, and ovaries are positively immunostained for SP-D (7). SP-D has been shown to bind to different strains of HIV-1 (BaL and IIIB) at pH 7.4 (physiological) and 5.0 similar to the pH found in the female urogenital tract (8). Glycoprotein gp120, a highly conserved mannosylated oligosaccharide present on the envelope of HIV-1 virion, plays an important role in the viral entry and facilitates viral replication by activating the NF-κB pathway. SP-D has been shown to bind gp120 of various strains of HIV-1 and prevent HIV-1 interaction with CD4 receptor on T cells, thereby inhibiting viral entry and replication (9, 10).

Another pattern recognition immune regulatory molecule, DC-SIGN/CD209, a type-II transmembrane protein of 44 kDa present on dendritic cell (DC) surface (11), plays a major role in mediating DC adhesion, migration, inflammation, and activation of T cell. DC-SIGN can serve as a route of immune escape for pathogens and tumors (12) and is a known receptor for many viruses, including HIV-1 and HIV-2. DC-SIGN is expressed by both mature and immature DCs in lymphoid tissues (11, 13), but not on follicular DCs, plasmacytoid DCs or CD1a^+^ Langerhans cells (14), monocytes, T cells, B cells, thymocytes, and CD34^+^ bone marrow cells. It is also expressed by polarized (M2) macrophages that infiltrate tumors (15), and on antigen-presenting cells such as macrophages, and in chorionic villi of placenta (16). Cells expressing DC-SIGN are located in T cell area of lymph nodes, tonsils, and spleen and dermal DCs in skin (CD14^+^ macrophages) (17). DC-SIGN expressing cells are seen in mucosal tissue of rectum (18) (with high antigen-presenting capacities), cervix, and uterus, in hepatic sinusoid and lymphatic sinus (19, 20).

HIV-1 virus, when exposed to genital and anal mucosal tissues, binds to DC-SIGN on tissue embedded DCs (21, 22) and gets transmitted to CD4^+^ T cells, activating adaptive immune response (23, 24). DC-SIGN facilitates HIV-1 transmission in both *cis* and *trans* fashion (25). Expression of DC-SIGN is regulated by IL-4 during monocyte–DC differentiation pathway, along with GM-CSF (26). TGF-β and IFNs are known to be inhibitors of DC-SIGN expression, and, thus, indirectly regulate HIV-1 transmission (26).

The interaction between HIV-1 and DC-SIGN takes place in the mucosal tract where SP-D is present. Since both SP-D and DC-SIGN can bind gp120, we set out to examine if interplay between these proteins can modulate DC-SIGN-mediated viral transfer of HIV-1. This view was further substantiated by observations that SP-D levels are increased in the broncho-alveolar fluid of HIV-1 patients (27); and recombinant forms of SP-D (rfhSP-D) can bind to gp120 of HIV-1, acting as a potent inhibitor of viral infection *in vitro via* inhibition of the interaction between CD4 and gp120 (10). In this study, we show, using recombinant forms of tetrameric and monomeric forms of DC-SIGN and its homolog, DC-SIGNR, that there is a protein–protein interaction between the two C-type lectins *via* CRD regions. They compete for binding to HIV-1 gp120, and thus, SP-D suppresses DC-SIGN mediated transfer of HIV-1 to CD4^+^ cells.

## Materials and Methods

### Recombinant Expression and Purification of Soluble Forms of Tetrameric and Monomeric DC-SIGN and DC-SIGNR

*E. coli* strain BL21 (λDE3) (Invitrogen, UK) was transformed with pT5T plasmid encoding DC-SIGN and DC-SIGNR sequences (inserted at the BamHI restriction site into plasmid construct) with and without multimerizing neck region. In the presence of neck region, the bacterial cells expressed tetrameric DC-SIGN and DC-SIGNR; without the neck region, the corresponding constructs produced monomeric forms of DC-SIGN and DC-SIGNR (28). *E. coli* strain BL21 (λDE3) cells containing ampicillin (50 µg/ml) (Sigma-Aldrich) resistant plasmids [except in the case of DC-SIGNR monomer expressing construct that was kanamycin (50 µg/ml) (Sigma-Aldrich) resistant] were subcultured overnight at 37°C. One liter LB medium containing ampicillin or kanamycin was inoculated with 10 ml of overnight bacterial culture and grown at 37°C until the OD_600_ reached 0.7, and then induced with 0.5 mM isopropyl β-d-1-thiogalactopyranoside (IPTG). After 3 h, the bacterial cells were centrifuged at 13,800 × *g* for 15 min to collect the bacterial pellet. Protein expression was analyzed *via* 12% SDS-PAGE.

The cell pellet was treated with 22 ml of lysis buffer, containing 100 mM Tris, pH 7.5, 0.5 M NaCl, lysozyme (50 µg/ml), 2.5 mM EDTA, pH 8.0, and 0.5 mM phenylmethylsulfonyl fluoride (PMSF), and left to stir for 1 h at 4°C. Cells were then sonicated for 10 cycles, each cycle of 30 sec with 2 min interval. The sonicated cell suspension was spun at 10,000 × *g* for 15 min at 4°C. The inclusion bodies, present in the pellet, were solubilized in 20 ml of 6 M urea, 10 mM Tris–HCl, pH 7.0, and 0.01% β-mercaptoethanol (β-ME) by rotating on a shaker for 1 h at 4°C. The mixture was then centrifuged at 13,000 × *g* for 30 min at 4°C and the supernatant was drop-wise diluted fivefold with loading buffer containing 25 mM Tris–HCl, pH 7.8, 1 M NaCl, and 2.5 mM CaCl_2_ with gentle stirring. This was then dialyzed against 2 l of loading buffer with three buffer changes every 3 h. Following further centrifugation at 13,000 × *g* for 15 min at 4°C, the supernatant was loaded onto a Mannan agarose column (5 ml; Sigma) pre-equilibrated with the loading buffer. The column was washed with five bed volumes of the loading buffer and the bound protein was eluted in 1 ml fractions using the elution buffer containing 25 mM Tris–HCl, pH 7.8, 1 M NaCl, and 2.5 mM EDTA. The absorbance was read at 280 nm and the peak fractions were frozen at −20. Purity of protein was analyzed by 15% w/v SDS-PAGE.

### Expression and Purification of rfhSP-D

*E. coli* BL21 (λDE3) pLysS containing plasmid pUK-D1 (containing cDNA sequences for 8 Gly–X–Y repeats, neck, and CRD region of human SP-D) was cultured in ampicillin (100 µg/ml) (Sigma-Aldrich) and chloramphenicol (50 µg/ml) (Sigma-Aldrich) at 37°C overnight. Expression and purification was carried out as described earlier (29, 30). Bacterial cells were grown until the OD_600_ reached 0.6 to 0.8, then induced with 0.4 mM IPTG and allowed to grow for an additional three hours. Cells were then pelleted *via* centrifugation and was re-suspended in 50 ml of lysis buffer (50 mM Tris–HCl, pH 7.5, 200 mM NaCl, 5 mM EDTA with freshly added 0.1 mM PMSF, and 100 µg/ml lysozyme) at 4°C for 1 h. The cell lysate was then sonicated at 4 kHz for 30 s with 2 min interval for 15 cycles. The sonicate was centrifuged at 13,800 × *g* for 15 min at 4°C to collect the rfhSP-D-rich pellet containing inclusion bodies. 25 ml of solubilization buffer (50 mM Tris–HCl, pH 7.5, 100 mM NaCl, 5 mM EDTA, 6 M urea) was used to re-suspend the pellet, and incubated at 4°C for 1 h. The dialysate was then centrifuged at 13,800 × *g*, at 4°C for 15 min, and the supernatant was dialyzed against solubilization buffer containing 4 M urea and 10 mM β-ME for 2 h at 4°C. The dialysis buffer was serially changed to solubilization buffer containing 2, 1, and 0 M urea at 4°C, 2 h each. Final dialysis was done in solubilization buffer containing 5 mM CaCl_2_ for 3 h to completely remove any traces of urea. The dialysate was centrifuged at 13,800 × g, 4°C for 15 min and the clear supernatant containing rfhSP-D was affinity-purified using maltosyl-agarose column (Sigma-Aldrich). The bound protein was eluted with solubilization buffer containing 10 mM EDTA, pH 7.5. Endotoxin levels were removed by passing the purified protein fractions through Polymyxin B column (Detoxi-Gel, Peirce & Warriner, UK) and the levels were measured using the Limulus Amebocyte Lysate Assay (BioWhitaker, UK). The endotoxin level was found to be <5 pg/μg rfhSP-D.

### SDS-PAGE and Far Western Blot Analysis

DC-SIGN and DC-SIGNR proteins were separated on a 12% (w/v) SDS-PAGE under reducing conditions. After electrophoresis, the polyacrylamide gels were stained with Coomassie Brilliant Blue. For the far western blotting, proteins were electro-transferred onto polyvinylidene difluoride (PVDF) membrane (Sigma) and blocked with 5% w/v milk in PBS. The membrane bound proteins were probed with rfhSP-D (5 μg/ml) for 2 h, followed by addition of anti-SP-D (1:1,000) (Medical Research Council Immunochemistry Unit, Oxford) polyclonal antibodies. The blot was then probed with Protein A-HRP Conjugate (1:1,000) (Sigma), followed by color development with diaminobenzidine as a substrate (Sigma-Aldrich, UK).

### ELISA

Microtitre wells were coated with DC-SIGN and DC-SIGNR proteins in carbonate/bicarbonate buffer, pH 9.6 in decreasing double dilutions (5–0.625 µg/well) in duplicates and left overnight at 4°C. The microtiter wells were blocked with 2% w/v BSA in PBS for 2 h at 37°C. The wells were then washed three times with PBS + 0.05% v/v Tween 20 and incubated with a constant concentration (2.5 µg) of rfhSP-D in 20 mM Tris–HCl, pH 7.5, 100 mM NaCl, 5 mM CaCl_2_ or 5 mM EDTA at 37°C for 1 h, followed by 1 h at 4°C. Following PBS + Tween 20 wash, the bound rfhSP-D was detected using anti-SP-D (1:5,000) polyclonal antibody and Protein A-HRP conjugate (1:5,000). Color was developed using *o*-Phenylenediamine (OPD) as a substrate and absorbance was measured at 490 nm.

### Competitive ELISA

The ability of rfhSP-D to compete with and DC-SIGN for binding to HIV-1 gp120 (Abcam; ab167715) gp120 was analyzed by competitive ELISA. Gp120 was coated at 250 ng/well in duplicates and left overnight at 4°C. Wells were blocked with 2% BSA in PBS for 2 h at 37°C. The wells were washed three times with PBS + 0.05% v/v Tween 20. A constant concentration of DC-SIGN tetramer (5 μg/ml) and decreasing concentrations of rfhSP-D (5 − 0.625 μg/well) in calcium buffer were added to the wells, which were subsequently probed with anti-DC-SIGN (1:5000) polyclonal antibodies. Following washes, the wells were incubated with Protein HRP conjugate (1:1,000). Color was developed using OPD as a substrate.

### Fluorescence Microscopy

Human embryonic kidney cells 293 (HEK 293), transfected with DC-SIGN construct (DC–HEK) (31), were grown and maintained in DMEM (Life technologies, UK) containing 10% v/v fetal calf serum, 2 mM l-glutamine, penicillin (100 U/ml)/streptomycin (100 µg/ml) (Thermo Fisher), and blasticidin (5 µg/ml) (Gibco). HEK 293 cells were grown and maintained in DMEM (Life technologies) containing 10% FBS. Both cell lines were grown under standard conditions (37°C, 5% v/v CO_2_) until 80–90% confluency was reached. HEK 293 and DC–HEK cells (0.5 × 10^5^) were grown on the coverslips in a 24-well plate (Nunc) overnight to perform three different sets of immunofluorescence experiments; DC-SIGN expression (primary antibody: rabbit anti-DC-SIGN, 1:500 and secondary antibody: anti-rabbit/CY3, 1:500, Thermo Fisher), rfhSP-D (10 µg/ml) binding to DC-SIGN (primary antibody: monoclonal anti-SP-D, 1:500 and secondary antibody: anti-mouse conjugated/CY5, 1:500, Abcam) and co-localization of DC-SIGN and rfhSP-D (primary antibodies: anti-DC-SIGN polyclonal and anti-SP-D monoclonal, 1:500 and secondary antibodies: anti-rabbit/CY3 and anti-mouse/FITC, 1:500) on the cell surface of DC–HEK cells. HEK 293 cells were used as a control for all experiments and DC–HEK cells were incubated with secondary antibody alone as an additional control. Hoechst (1:10,000, Thermo Fisher) was used to stain the nucleus for all the staining experiments. The cells were incubated for 1 h with primary antibody followed by 1 h incubation with secondary antibodies as described earlier with three times phosphate-buffered saline (PBS, Thermo Fisher) washes between each step. For rfhSP-D binding with DC-SIGN analysis, the rfhSP-D was incubated with the cells for 1 h at 4°C. The cells were fixed with 4% paraformaldehyde (Sigma) before mounting on the coverslips to visualize under a HF14 Leica DM4000 microscope.

### Viral Transfer Assay

Pooled human peripheral blood mononuclear cells (PBMCs) (HiMedia Laboratories, India) were stimulated in RPMI 1640 medium (Sigma-Aldrich) containing 10% v/v FBS, 1% Penicillin–Streptomycin and 5 µg/ml phytohemaglutinin (PHA) and 10 U/ml of rhIL-2 (Gibco) for 24 h. PHA/IL-2 was washed off and activated PBMCs were cultured further in complete RPMI medium. For the experiment, DC–HEK cells were grown in a 12-well tissue culture plate until 80% confluence in complete DMEM/F12 (Sigma-Aldrich, USA) containing 10% FBS (Gibco) and blasticidin. Indicated concentrations of rfhSP-D containing 5 mM CaCl_2_ was added to each well and incubated for 2 h to allow binding to DC-SIGN. The wells without rfhSP-D were used as controls. Excess protein was removed, and cells were challenged for 1 h with 5 ng/ml p24 of HIV-1 SF-162 strain (kindly provided by Dr. Jay Levy, AIDS Program, National Institutes of Health, USA). 5 mM EDTA was added along with the virus in EDTA controls. Unbound virus was washed off and DC–HEK cells were cocultured with PHA/IL-2 activated PBMCs for 24 h to facilitate transfer. PBMCs along with the medium were then separated (siphoned off) from the DC–HEK monolayer and were transferred to fresh plates. They were cultured further in RPMI 1640 medium containing 10% FBS for 7 days, and viral titers were determined in supernatants on day 4 and 7 using HIV-1 p24 antigen ELISA kit (XpressBio Life Science Products, Frederick, MD, USA).

### Molecular Modeling and Bioinformatics

The crystal structures of trimeric human lung SP-D (PDB ID: 1PW9), CD4 bound to HIV-1 envelope glycoprotein gp120 (PDB ID:1GC1) and homo 10-mer DC-SIGN complexed with sugars (PDB ID:1K9I) were retrieved from Protein Data Bank. The tetrameric form of non-glycosylated DC-SIGN was used for docking studies as this structure was found to bind to rfhSP-D *in vitro* experiments. DC-SIGN tetramer was docked to CD4 already bound to HIV-1 envelope glycoprotein gp120 (PDB ID: 1GC1) using Patch Dock server with default parameters.

The CRD-mediated protein–protein interaction between trimeric SP-D and tetrameric DC-SIGN, as observed in this study was further examined by docking these two molecules using ZDOCK algorithm of Discovery Studio (Accelrys Inc.). The best pose of these two molecules was subsequently docked into gp120 using Patch Dock server. The shortlisted poses from PatchDock and ZDOCK were further refined using Fire Dock and RDOCK, respectively.

## Results

### rfhSP-D and DC-SIGN Can Interact with Each Other *via* Their C-Type Lectin Domains

**Figure 1:**
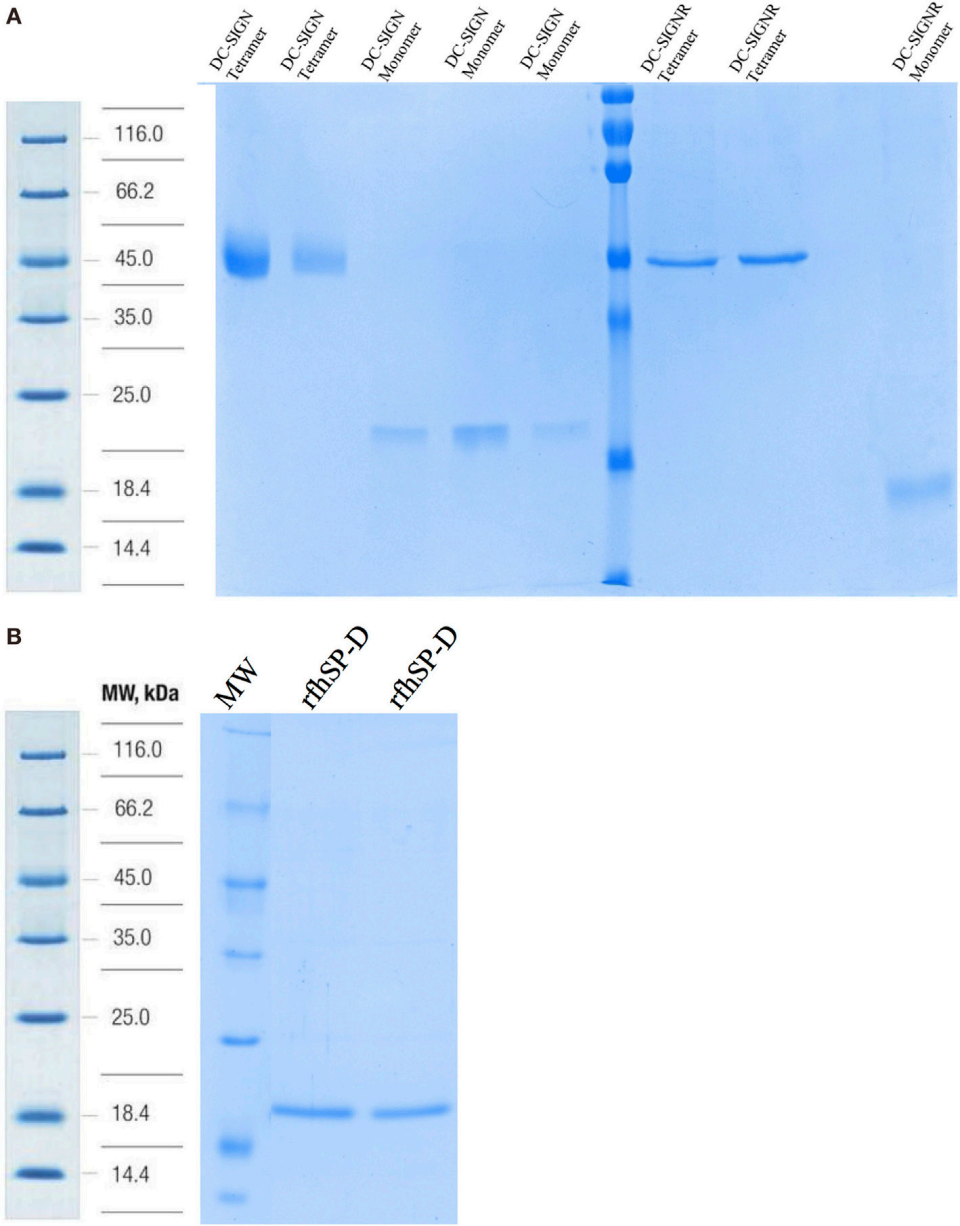
SDS-PAGE analysis of purified recombinant forms of DC-SIGN, DC-SIGNR, and recombinant forms of SP-D (rfhSP-D). **(A)** 12% SDS-PAGE of affinity-purified tetrameric and monomeric forms of DC-SIGN and DC-SIGNR under reduced conditions. **(B)** 12% v/v SDS-PAGE of affinity-purified rfhSP-D.

**Figure 2:**
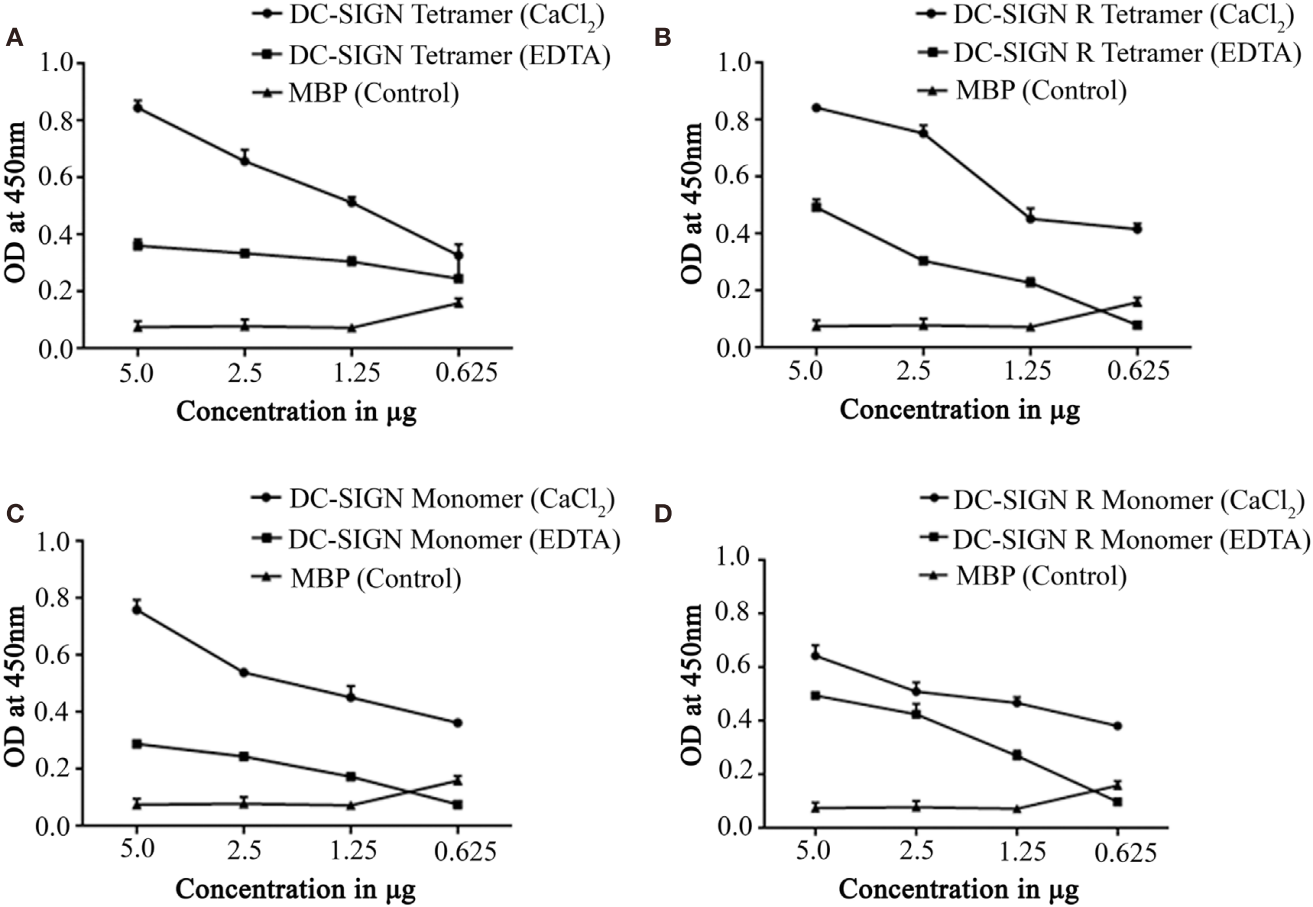
Direct binding ELISA showing interaction between recombinant forms of SP-D (rfhSP-D) and DC-SIGN/DC-SIGNR. DC-SIGN tetramer **(A)**, DC-SIGNR tetramer **(B)**, DC-SIGN monomer **(C)**, and DC-SIGNR monomer **(D)** were coated at decreasing double dilutions from 5 to 0.625 µg/well and then probed with 2.5 µg of rfhSP-D in either in calcium or EDTA buffer. The binding was detected using anti-human surfactant protein D polyclonal antibodies (1:5,000 dilutions). The data represent mean and SD values of at least five experiments.

**Figure 3:**
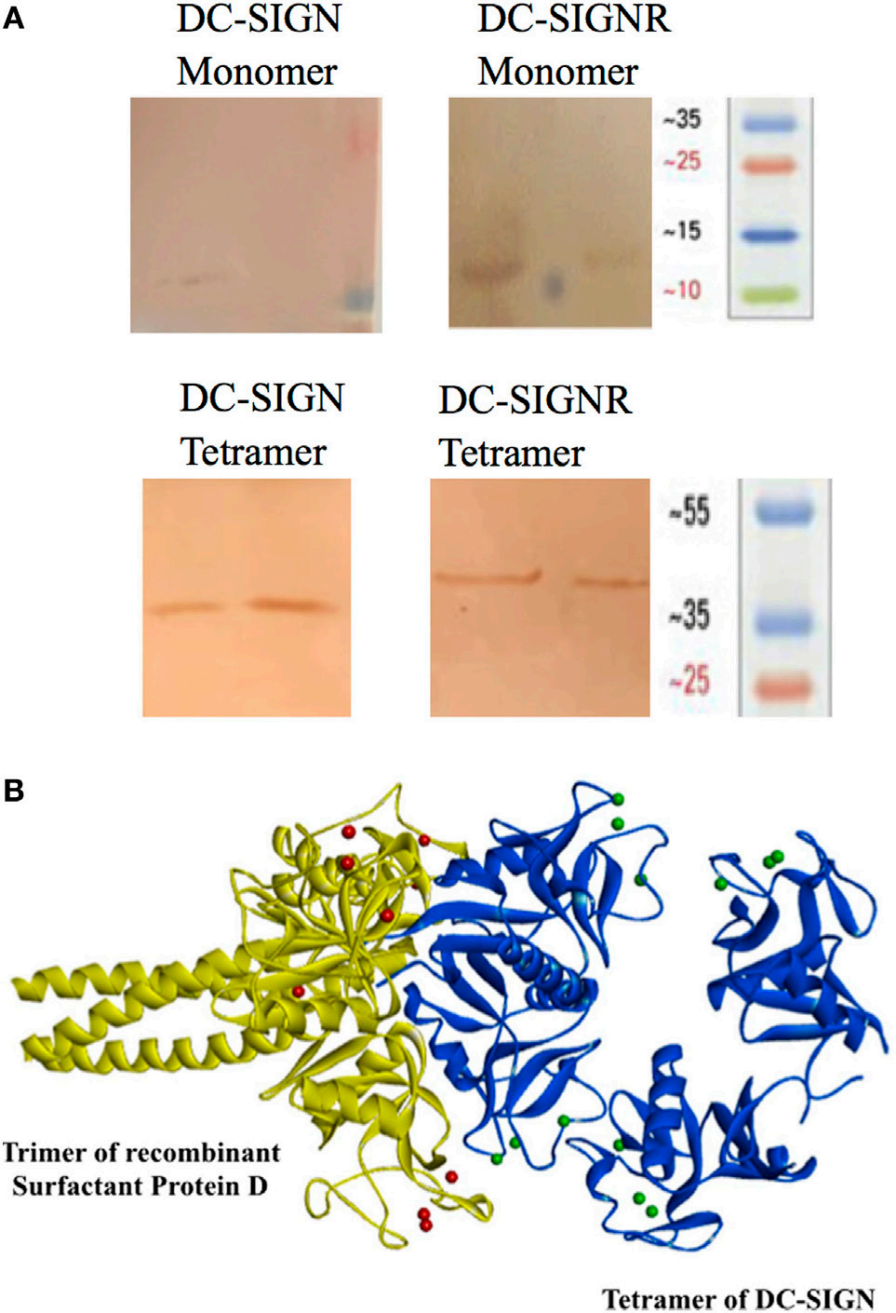
Far western blot to detect binding of recombinant forms of SP-D (rfhSP-D) to PVDF bound DC-SIGN and DC-SIGNR. **(A)** Tetrameric and monomeric variants of DC-SIGN and DC-SIGNR were run on a SDS-PAGE and were transferred to a PVDF membrane followed by incubation with 5 µg/ml rfhSP-D and then probed with anti-SP-D polyclonal antibody. **(B)** Docked structure of trimeric surfactant protein D (SP-D) (yellow cartoon) and tetrameric DC-SIGN (blue cartoon). The two molecules interact *via* their carbohydrate recognition domains.

Structurally, DC-SIGN is composed of an extracellular domain (ECD) which exists as a tetramer, stabilized by an N-terminal α-helical neck region, followed by a CRD. DC-SIGN and DC-SIGNR comprising of the entire ECD (tetramer) (Figure 1A) and the CRD region alone (monomer) (Figure 1A) were expressed in *E. coli* and affinity-purified on Mannose-agarose (28). The CRD regions of DC-SIGN and SIGNR bound mannose weakly as majority of the proteins appeared in the flow through. The ECD domains of both DC-SIGN and DC-SIGNR bound to mannose with much greater affinity in the presence of Ca^2+^ and eluted with EDTA. A recombinant form of human SP-D, containing 8 Gly–X–Y repeats of the collagen, neck, and CRD regions were expressed and purified as homotrimeric molecules, as described earlier (29, 30) (Figure 1B). Tetrameric and monomeric forms of DC-SIGN and DC-SIGNR were checked for their respective interactions with rfhSP-D *via* ELISA (Figure 2), which showed a calcium- and dose-dependent interaction between the two lectins; tetrameric forms bound rfhSP-D better than the monomers. This was confirmed by a far western blot (Figure 3A), which revealed that rfhSP-D was able to bind to DC-SIGN and DC-SIGNR proteins immobilized on PVDF membrane. The CRD-mediated protein–protein interaction between trimeric SP-D and tetrameric DC-SIGN was further studied by docking these two molecules. The docked pose showed that the two molecules likely interact *via* their CRD regions (Figure 3B).

### rfhSP-D:DC-SIGN Interaction Leads to Competition for Binding to HIV-1 gp120

**Figure 4:**
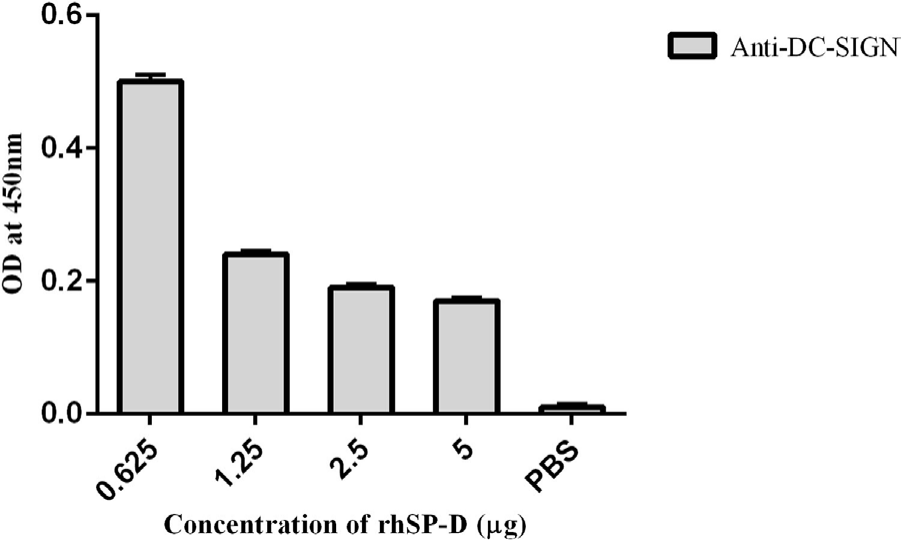
Competitive inhibition ELISA to show that recombinant form of SP-D (rfhSP-D) inhibits DC-SIGN binding to immobilized HIV-1 gp120. HIV-1 gp120 trimer (250 ng per well) was first coated to which 5–0.625 µg/well of rfhSP-D and a constant concentration (5 µg/well) of DC-SIGN tetramer were added. Bound DC-SIGN tetramer was detected by anti-DC-SIGN polyclonal antibodies. Protein A-HRP conjugate (1:1,000) was used to detect the antibodies bound and color was developed using *o*-Phenylenediamine. 0 in the graph represents the control where only PBS was used instead of gp120, and the experiments were repeated three times.

To examine if rfhSP-D can inhibit the binding of DC-SIGN to gp120, we carried out a competitive ELISA. As expected, both rfhSP-D and DC-SIGN tetramer bound gp120 in a dose- and calcium-dependent manner (data not shown) (32). In order to assess a likely interference by rfhSP-D in DC-SIGN: gp120 interaction, a constant concentration of DC-SIGN tetramer was used against different concentrations of rfhSP-D and added to solid-phase gp120 (Figure 4). With increasing concentration, rfhSP-D was able to inhibit DC-SIGN-gp120 interaction, suggesting that the binding sites on these two C-type lectins for gp120 may be overlapping.

### rfhSP-D Co-Localizes with DC-SIGN on the Surface of Transfected HEK 293 Cells

**Figure 5:**
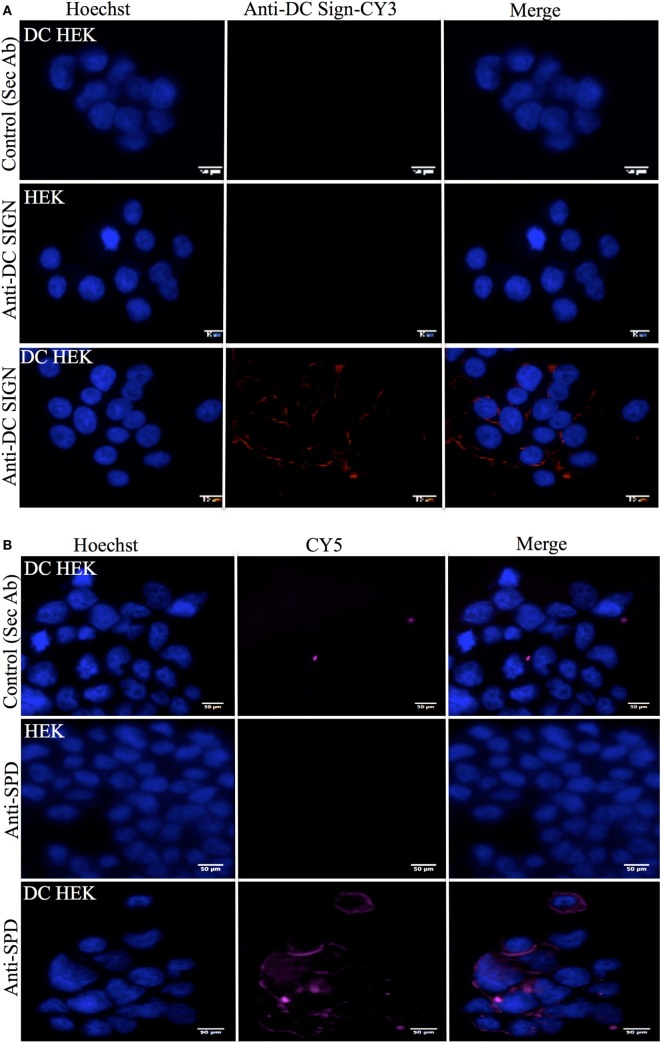

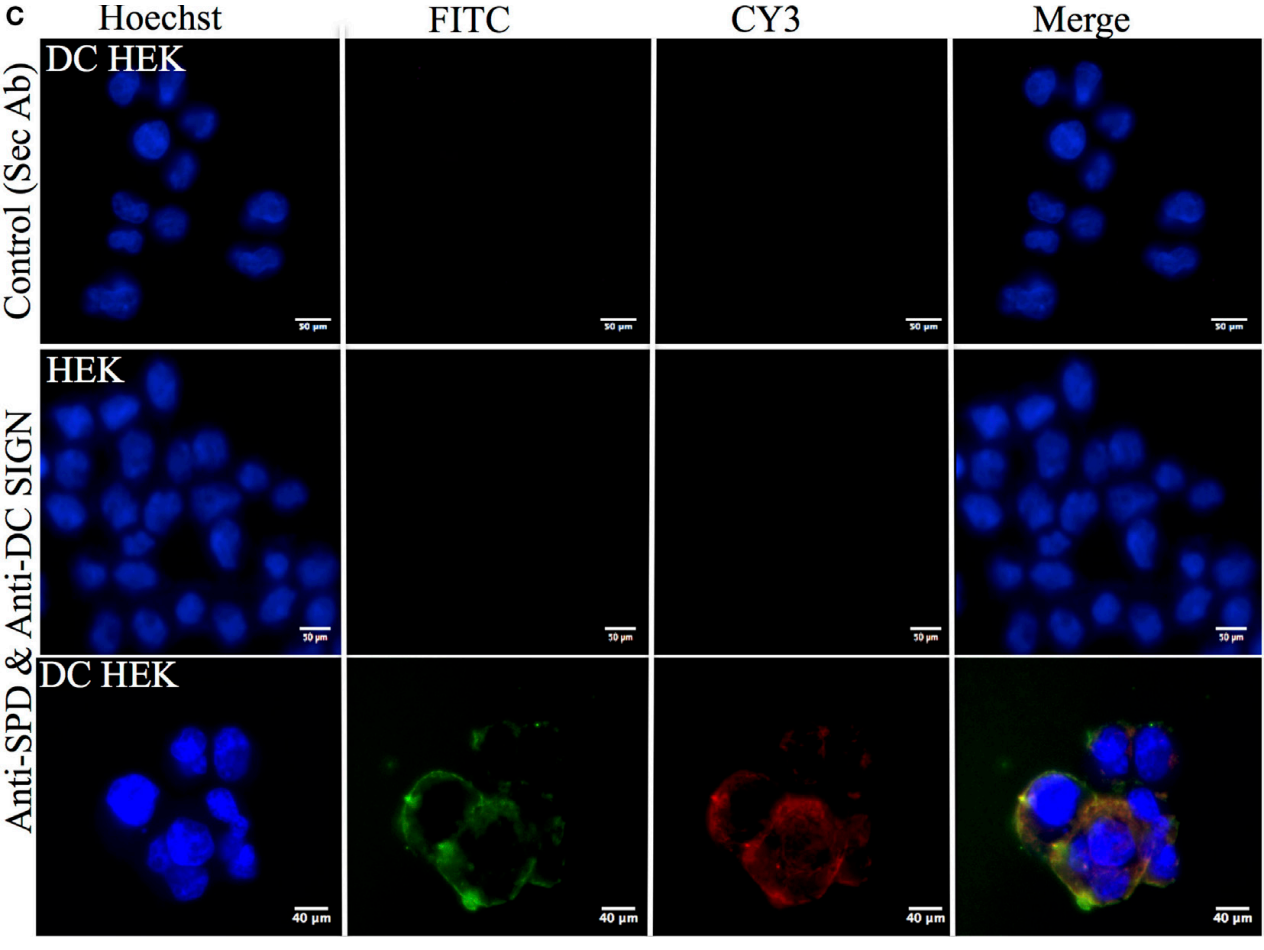
Immunofluorescence microscopy to show recombinant forms of SP-D (rfhSP-D) binding to DC-SIGN on the surface of the human embryonic kidney (HEK) cells transfected with DC-SIGN construct (DC–HEK cells). **(A)** DC–HEK cells incubated with anti-rabbit/CY3 did not show DC-SIGN expression (control). DC–HEK and HEK cells incubated with anti-DC-SIGN followed by anti-rabbit conjugated with CY3 showed the DC-SIGN expression in DC–HEK cells only and not HEK cells. Hoechst was used to stain the nucleus. **(B)** Analysis of rfhSP-D binding to DC-SIGN on the DC–HEK cells *via* immunofluorescence. DC–HEK cells incubated with anti-SP-D for 1 h and then probed with anti-mouse/CY5 did not show binding. DC–HEK cells incubated with rfhSP-D (5 µg/ml) for 1 h, followed by anti-SP-D for 1 h and then anti-mouse/CY5 showed the binding on the cell surface. **(C)** DC-SIGN expression and rfhSP-D binding co-localization analysis *via* immunofluorescence microscopy. DC–HEK cells incubated with secondary antibodies only (anti-mouse/FITC and anti-rabbit/FITC) for 1 h did not show immunofluorescence. DC–HEK and HEK cells incubated with rfhSP-D for 1 h prior to incubation anti-SP-D monoclonal and anti-DC-SIGN polyclonal for 1 h followed by anti-mouse/FITC and anti-rabbit/CY3 for 1 h showed co-localization for rfhSP-D binding and DC–HEK expression.

Human embryonic kidney cells transfected with DC-SIGN construct (DC–HEK cells) were shown to express DC-SIGN *via* immunofluorescence microscopy. The DC-SIGN expression seen on the cell surface on DC–HEK cells was evenly distributed, as compared to HEK 293 cells, which were used as a control, using anti-DC-SIGN polyclonal antibody (Figure 5A). DC–HEK cells, incubated with secondary antibody alone, did not show any expression (Figure 5A). rfhSP-D binding was visible on the cell surface of DC–HEK cells, whereas rfhSP-D binding could not be detected in either HEK 293 cells or DC–HEK cells incubated with secondary antibody alone as controls (Figure 5B). rfhSP-D and DC-SIGN co-localized on the HEK cell surface transfected with DC-SIGN construct (Figure 5C).

### rfhSP-D Inhibits DC-SIGN-Mediated Viral Transfer to PBMCs in a Dose-Dependent Manner

**Figure 6:**
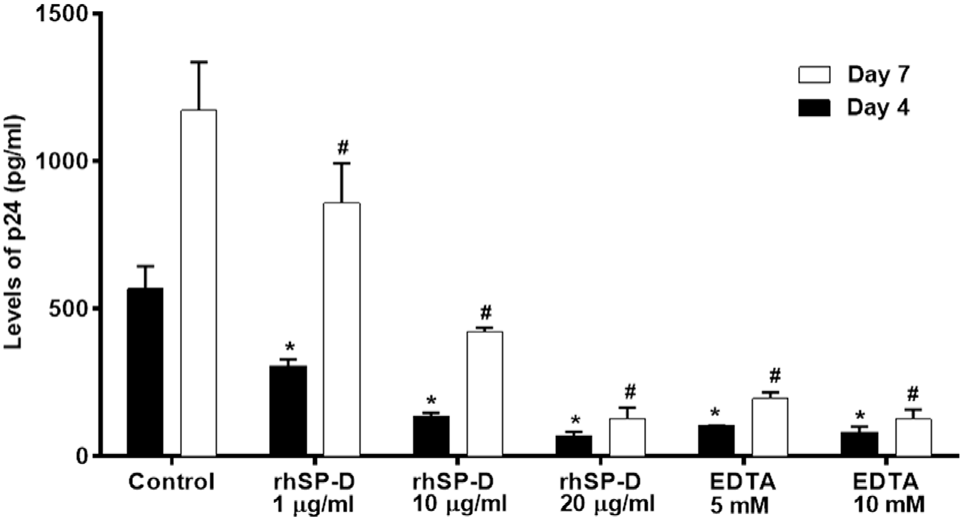
DC-SIGN-mediated HIV-1 transfer assay. DC–HEK cells were grown in a 12-well plate until 80% confluence. 20, 10, and 1 µg/ml of recombinant forms of SP-D (rfhSP-D) concentrations were added to the cells and incubated for 2 h for binding. Unbound protein was removed and cells were challenged with 2.5 ng/ml p24 of HIV-1 (SF-162 strain) for 1 h (to bind to DC-SIGN). After 1 h, unbound virus was washed off and cells were cocultured with phytohemaglutinin-activated peripheral blood mononuclear cells (PBMCs) for 24 h. This allows the DC-SIGN captured virus to be transferred to CD4^+^ cells, where virus will multiply. PBMCs were separated from the monolayer and cultured separately for 4 days to determine viral titer.

To understand whether interaction between rfhSP-D and DC-SIGN impacted upon DC-SIGN-mediated HIV-1 transfer to T cells, we performed a coculture assay using DC–HEK cells and mitogen-activated PBMCs. Presence of rfhSP-D led to a significantly (*p* < 0.005) reduced HIV-1 p24 levels in day 4 and day 7 PBMC culture supernatants in a dose-dependent manner (Figure 6). This suggested that, in presence of rfhSP-D, the viral uptake by DC–HEK was significantly inhibited resulting in reduced transfer and replication of HIV-1 in PBMC cultures. It is likely that rfhSP-D may have occupied sites on both DC-SIGN as well as HIV-1 gp120 that resulted in reduced DC-SIGN interaction with HIV-1 gp120. EDTA significantly inhibited DC–HEK-mediated viral transfer, as reported previously (33).

### Bioinformatics Analysis Revealed That HIV-1 gp120 and rfhSP-D May Occupy the Same Site on the CRDs of DC-SIGN

**Figure 7:**
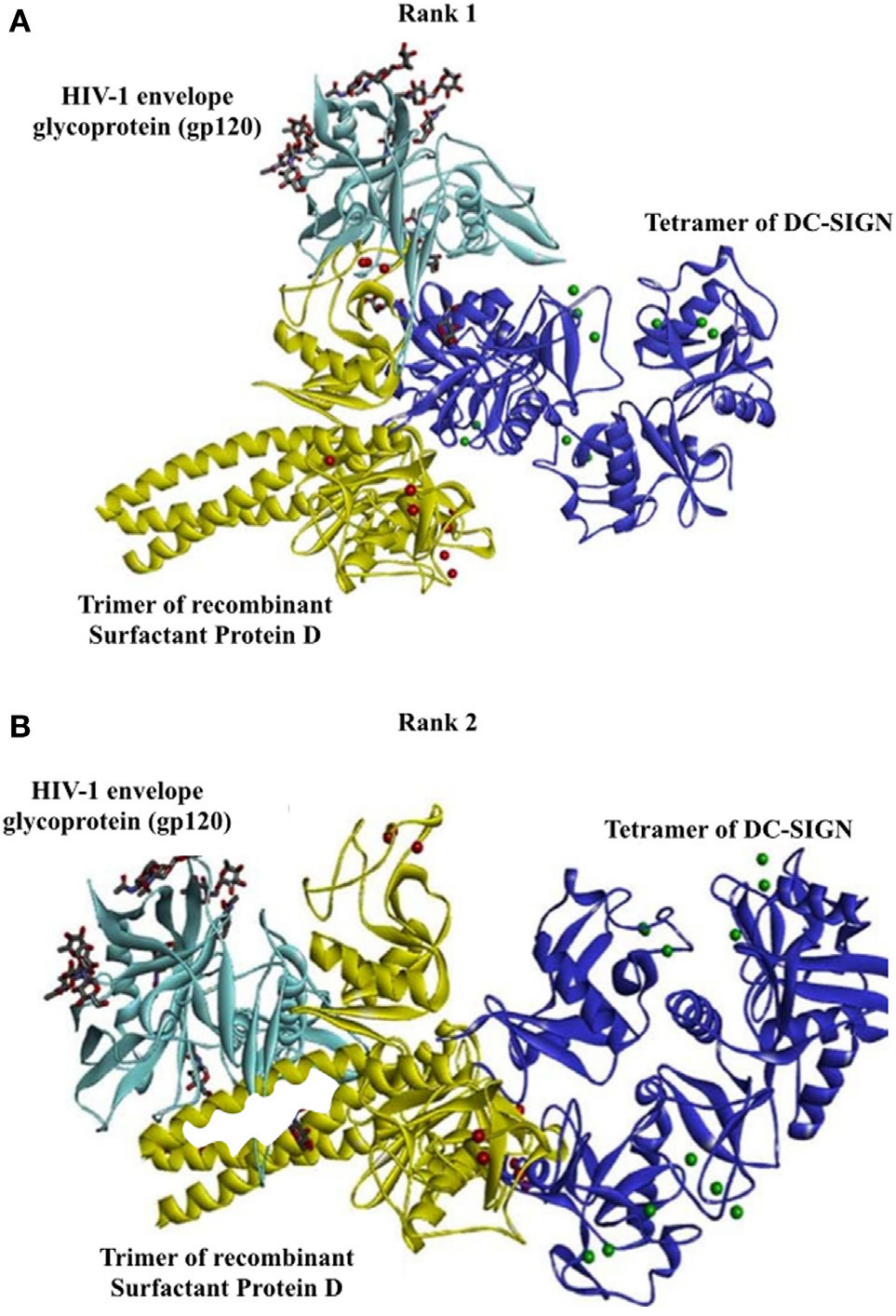
Two poses suggesting that HIV-1 gp120 and recombinant forms of SP-D possibly occupy the same site on carbohydrate recognition domain of DC-SIGN. Docked structures of surfactant protein D trimer (yellow cartoon and calcium ions as red spheres) complexed with DC-SIGN tetramer (blue cartoon and calcium ions as green spheres) and HIV-1 envelope glycoprotein, gp120 (cyan cartoon). The sugars present in gp120 are shown as sticks. The calcium ions of DC-SIGN are represented as green spheres. **(A)** Rank no. 1. **(B)** Rank no. 2.

**Figure 8:**
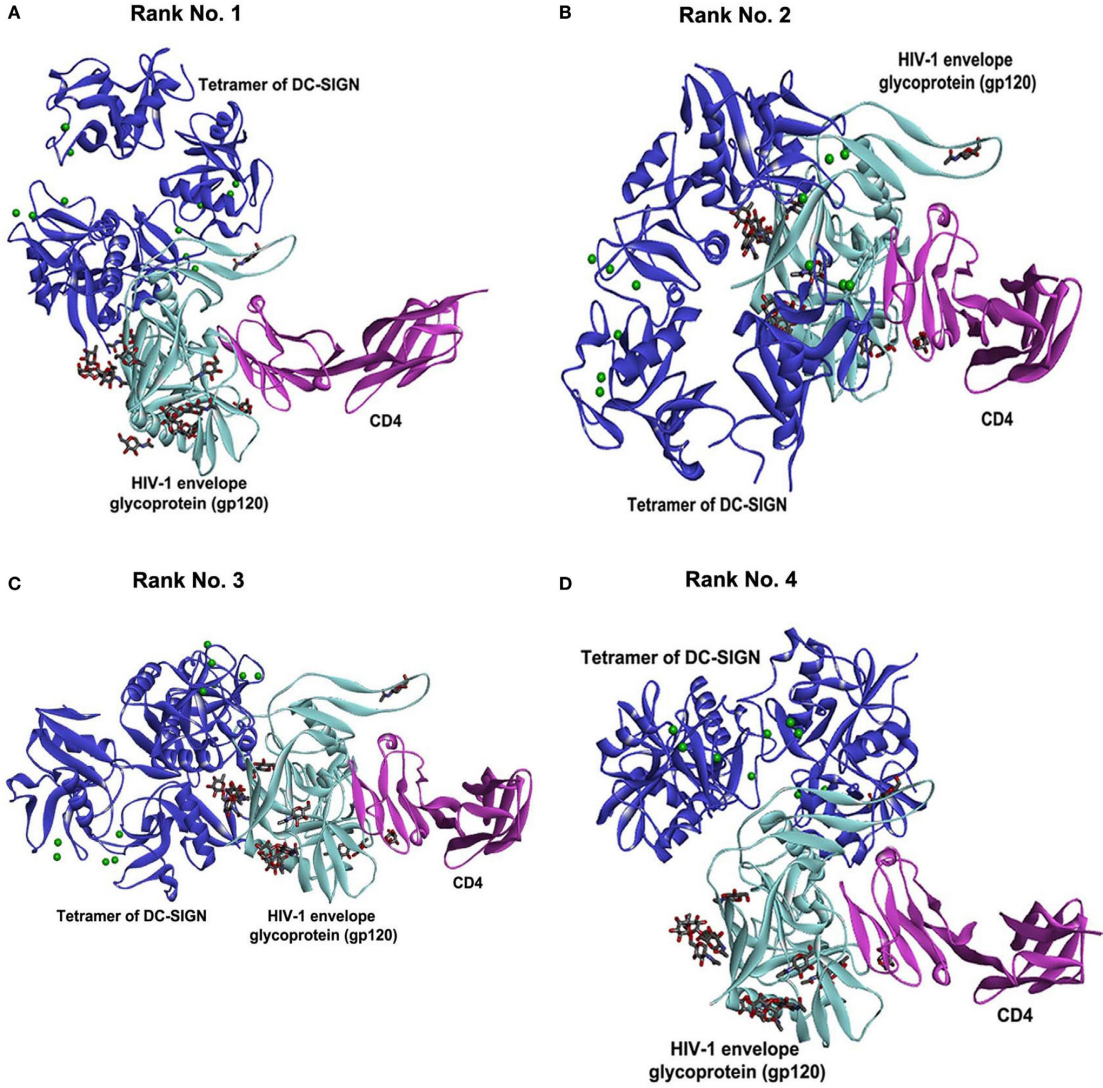
Known interaction of gp120 with DC-SIGN followed by docking with CD4. Selected docked poses of tetrameric DC-SIGN (blue cartoon) and HIV-1 envelope glycoprotein gp120 (cyan cartoon) bound to CD4 (pink cartoon). The sugars present in gp120 are shown as sticks. **(A)** Rank no. 1. **(B)** Rank no. 2. **(C)** Rank no. 3. **(D)** Rank no. 4.

To support our hypothesis that DC-SIGN once bound to rfhSP-D may not interact with gp120, we performed *in silico* analyses. The best-docked pose of rfhSP-D and DC-SIGN was subsequently docked to gp120 using Patch Dock server. The shortlisted poses from Patch Dock and ZDOCK were further refined using Fire Dock and RDOCK, respectively. Two poses appear to suggest that HIV-1 gp120 and rfhSP-D possibly occupy the same site on the CRD of DC-SIGN (Figure 7). Thus, in the presence of rfhSP-D, it is likely that interaction of DC-SIGN with gp120 could be inhibited. To validate our bioinformatics strategy, we evaluated the known interaction of gp120 with DC-SIGN followed by docking with CD4. DC-SIGN binds to gp120 at a site distant from its CD4 binding site, and hence, DC-SIGN-bound HIV-1 possibly interacts with CD4 for viral transmission (Figure 8). The global energy of these docked complexes has also been presented (Table 1).

**Table 1:** Energy for docked complexes of DC-SIGN and gp120 bound to CD4 refined using FireDock.

Rank no.	Global energy (kcal/mol)
1	−27.01
2	−21.83
3	−11.99
4	−10.94

## Discussion

In this study, we report, for the first time, an interaction of DC-SIGN and SP-D, two C-type lectins and pattern recognition receptors; both proteins are known to bind to HIV-1 gp120. We demonstrate that this interaction involves their CRD domains, which is relevant in inhibiting DC-SIGN-mediated HIV-1 trans-infection of CD4^+^ T cells. Interaction of HIV-1 gp120 with DC-SIGN not only increases the affinity of gp120 for CD4 (34) but also leads to a productive infection *via* reactivation of provirus involving NF-κB pathway (35, 36). This interaction also results in downregulation of Nef-induced release of IL-6 (37) and leads to Ask-1-dependent activation leading to induction of apoptosis of human DCs (38). Simultaneous binding of rfhSP-D to both gp120 and DC-SIGN, thus, may result in blockade of DC-SIGN-mediated viral transmission and inhibition of replication.

Structure–function studies have revealed that the CRD region of DC-SIGN is the specific ligand-binding site that is reliant on the neck region within the extracellular domain (ECD) (39). This notion was validated in our binding ELISA type assays when we used the tetrameric forms of DC-SIGN and DC-SIGNR (comprising of the ECD and CRD region) as well as the monomeric forms, which only consist of the CRD region. The binding studies involving rfhSP-D highlighted that multimeric forms of DC-SIGN and DC-SIGNR bind better, not surprisingly, due to multivalent nature of interactions. Since DC-SIGN promotes HIV-1 infection, we examined if rfhSP-D by virtue to its ability to bind gp120 as well as DC-SIGN can potentially interfere with HIV-1 (40–42). We also included DC-SIGNR (DC-SIGN-Related), a homolog of DC-SIGN, in our study. DC-SIGNR, expressed on endothelium including liver sinusoidal, lymph node sinuses, and placental capillary, can also bind gp120 to facilitate HIV-1 viral infection (43).

The current study provides the first evidence that DC-SIGN is a novel immune receptor or adaptor for the CRD region of SP-D, modulating the HIV-1 infection. Interaction of gp120 and rfhSP-D is calcium dependent as reported earlier (8–10). Tetrameric DC-SIGN also efficiently binds gp120 in a dose-dependent manner, which is not significantly inhibited in presence of sugars similar to previous reports (28, 44). The recombinant rfhSP-D has been shown to inhibit the gp120-CD4 interaction (10) while, DC-SIGN-bound trimeric gp140 interacts with CD4 more avidly (34). *In vitro* competitive assays and the bioinformatics analysis confirmed that rfhSP-D and DC-SIGN compete for gp120. The reduced p24 levels confirmed that rfhSP-D significantly inhibits the DC-SIGN mediated viral transfer.

The rfhSP-D molecule (a recombinant fragment of human SP-D comprising homotrimeric C-type lectins), with part of collagen region, α-helical coiled-coil neck, and CRD region, has been extensively studied *via in vitro, in vivo*, and *ex vivo* experiments. In a number of studies, rfhSP-D has worked at par with full-length SP-D, as evident from its ability to be therapeutic in murine models of allergic bronchopulmonary aspergillosis (45, 46), invasive pulmonary aspergillosis (45), and dust mite allergy (47). It can also induce apoptosis in activated eosinophils (29, 48) and PBMCs (49). Thus, rfhSP-D is an excellent well-tested therapeutically active molecule.

Mannose-binding lectin, another serum collectin, is also known to inhibit DC-SIGN-mediated trans-infection of HIV-1 T cells (50) whereas SP-A and SP-D facilitate this transfer (8, 51). Madsen et al. incubated SP-D-HIV-1 complexes with immature monocyte-derived DCs and demonstrated increased viral uptake and transfer from DCs to PM-1 cells. However, the assay system employed in the two studies (Madsen and ours) significantly differed, thus the observed variation in the results. Further studies in appropriate animal models will help to determine the overall effects of SP-D and DC-SIGN binding during virus infections. Our findings have revealed a new phenomenon in SP-D-mediated viral transfer through DCs as rfhSP-D occupies similar sites as gp120 on DC-SIGN. Hence, pre-incubation of rfhSP-D may have occupied gp120-binding site on DC-SIGN (displacement of gp120 *via* ELISA and *in silico* analysis), resulting in poor uptake. This must have resulted in reduced transfer of viral particles to activated PBMCs, adding another aspect to rfhSP-D-mediated anti-HIV activity.

To summarize, rfhSP-D has the ability to directly inhibit the viral entry by interacting with gp120 and to significantly inhibit the DC-SIGN-mediated viral transfer. Importantly, these molecular interactions inhibit the immunomodulation mediated by gp120 and DC-SIGN further disfavoring the HIV-1 pathogenesis. DC-SIGN binding to SP-D could be one of the ligand–receptor interactions that in turn could play a major role in the inhibition of viral entry. Further, *in vivo* assays and clinical trials can elucidate the physiological conditions for therapeutic purposes against the infection.

## Author Contributions

ED-M, DM, HP, and AK carried out crucial set of experiments; supporting experiments were done by AS, VM, ST, BN, and MA-M; TM supervised critical infection assays; UK led the project and wrote the manuscript with due help from ED-M, HP, and TM.

## Conflict of Interest Statement

The authors declare that the research was conducted in the absence of any commercial or financial relationships that could be construed as a potential conflict of interest.
